# Potential Benefits of Combined Statin and Metformin Therapy on Resistance Training Response in Older Individuals

**DOI:** 10.3389/fphys.2022.872745

**Published:** 2022-04-14

**Authors:** Douglas E. Long, Kate Kosmac, Cory M. Dungan, Marcas M. Bamman, Charlotte A. Peterson, Philip A. Kern

**Affiliations:** ^1^ Department of Physical Therapy and Center for Muscle Biology, College of Health Sciences, University of Kentucky, Lexington, KY, United States; ^2^ Florida Institute for Human and Machine Cognition, Pensacola, FL, United States; ^3^ Center for Exercise Medicine and Department of Cell, Developmental, and Integrative Biology, University of Alabama at Birmingham, Birmingham, AL, United States; ^4^ Department of Internal Medicine, Division of Endocrinology, Barnstable Brown Diabetes and Obesity Center, University of Kentucky, Lexington, KY, United States

**Keywords:** statin, metformin, muscle hypertrophy, cellular features, resistance training

## Abstract

Metformin and statins are currently the focus of large clinical trials testing their ability to counter age-associated declines in health, but recent reports suggest that both may negatively affect skeletal muscle response to exercise. However, it has also been suggested that metformin may act as a possible protectant of statin-related muscle symptoms. The potential impact of combined drug use on the hypertrophic response to resistance exercise in healthy older adults has not been described. We present secondary statin analyses of data from the MASTERS trial where metformin blunted the hypertrophy response in healthy participants (>65 years) following 14 weeks of progressive resistance training (PRT) when compared to identical placebo treatment (*n* = 94). Approximately one-third of MASTERS participants were taking prescribed statins. Combined metformin and statin resulted in rescue of the metformin-mediated impaired growth response to PRT but did not significantly affect strength. Improved muscle fiber growth may be associated with medication-induced increased abundance of CD11b+/CD206+ M2-like macrophages. Sarcopenia is a significant problem with aging and this study identifies a potential interaction between these commonly used drugs which may help prevent metformin-related blunting of the beneficial effects of PRT.

**Trial Registration:** ClinicalTrials.gov, NCT02308228, Registered on 25 November 2014.

## Introduction

With an ever-increasing aged demographic, there continues to be a need for using preventative rather than reactive strategies to delay age-associated chronic disease, prolong quality of life, and extend healthspan, defined as the period of life void of debilitating pathology. Physical exercise is one unchallenged strategy to counter aging processes, such as the loss of muscle mass and strength leading to frailty, and prevent or manage chronic ailments (Bushman, 2020; McGee and Hargreaves, 2020). Exercise is as effective as prescribed medication (Naci and Loannidis, 2013) but less than 25% of the population meets physical activity recommendations provided by the Centers for Disease Control and Prevention (CDC).

The repurposing of common medications, especially those which have preventative indications for diabetes and cardiovascular events, such as metformin and statins, have been the focus of new clinical investigations aimed at healthspan (Kaeberlein, 2018). Metformin and statins are consistently ranked within the top five most prescribed medications, with almost 200 million prescriptions combined in the United States alone. However, experimental animal models show mixed results in improving healthspan with metformin (Smith et al., 2010; Martin-Montalvo et al., 2013; Wei et al., 2020), while statin translational trials offer little to no evidence for effects on healthspan in otherwise healthy individuals (Mansi et al., 2016; Zhou et al., 2020). There is a clear need to better understand specific “anti-aging” pharmaceuticals and their potential impact in individuals without an indication for their use as well as those already leading healthy lifestyles through exercise (Miller and Thyfault, 2020).

We recently completed the “Metformin to Augment Strength Training Effective Response in Seniors” (MASTERS) trial. This was a placebo controlled, double-blinded, progressive resistance training (PRT) study with randomized use of metformin in healthy older persons to examine the drug’s potential effects on muscle hypertrophy, inflammation, and other responses to PRT (Long et al., 2017; Walton RG. et al., 2019). Approximately 1/3 of the participants in this trial were also taking therapeutic doses of statins, and this report presents secondary analyses of this subgroup compared to those not taking statins.

## Methods

Previous studies have shown that overall, PRT is effective at increasing muscle mass and strength in older individuals, but there are a significant number of individuals who respond poorly to PRT in exercise trials (Lavin et al., 2019). The goal of our MASTERS trial was to determine if metformin combined with PRT could benefit functional performance in individuals over the age of 65 by increasing muscle hypertrophy and strength gains, essentially eliminating the “non-responder” phenotype. Individuals participated at two locations; 1700 mg of either metformin or placebo was consumed daily over 16 weeks, which included a 2-week medication ramping for tolerance followed by 14 weeks of PRT, performed three times per week, optimally designed for muscle hypertrophy. Vastus lateralis muscle biopsies were obtained at baseline, following medication ramping, and following 14-weeks of PRT to assess short-term medication and long-term exercise-medication interaction on cellular and molecular muscle adaptations. The main findings of this study reported that metformin negatively impacted the muscle hypertrophic response to resistance training (Walton RG. et al., 2019). Complete details, methods, and subject demographics of those completing the MASTERS trial have been reported elsewhere (Long et al., 2017; Walton RG. et al., 2019; Long et al., 2021).

### Statistics

Statistical analyses were performed using JMP Pro 14.0 software. Statistical evaluation of groups was performed to find possible baseline differences between groups that may contribute to hypertrophy and functional change using Chi-Square for nominal values and ANOVA with multiple comparison Tukey’s post hoc testing. ANCOVA at baseline for fiber type frequencies, and 2 × 2 ANCOVA with Tukey’s post hoc testing was used for group response to PRT. Statistical significance was defined as *p* < 0.05. Covariates were determined for their relationships with the dependent variables (fCSA, strength, capillaries, macrophages) in question. In addition, sex was considered a confounder as more males were taking statins in our cohort than females and more females completed the exercise training protocol in the placebo group. Statin users were, not unexpectedly, more likely to be using antihypertensives and NSAIDS when compared to our non-statin groups. Different n’s across variables are the result of missing data or muscle biopsy integrity issues which are given within each figure.

### Study Approval

This study was approved by the University of Kentucky institutional review board (IRB 14-0330) and the University of Alabama at Birmingham institutional review board (IRB F140722001) prior to any participants enrolling. Data and safety monitoring were provided by the UK CCTS DSMB on a quarterly basis.

## Results

**FIGURE 1:**
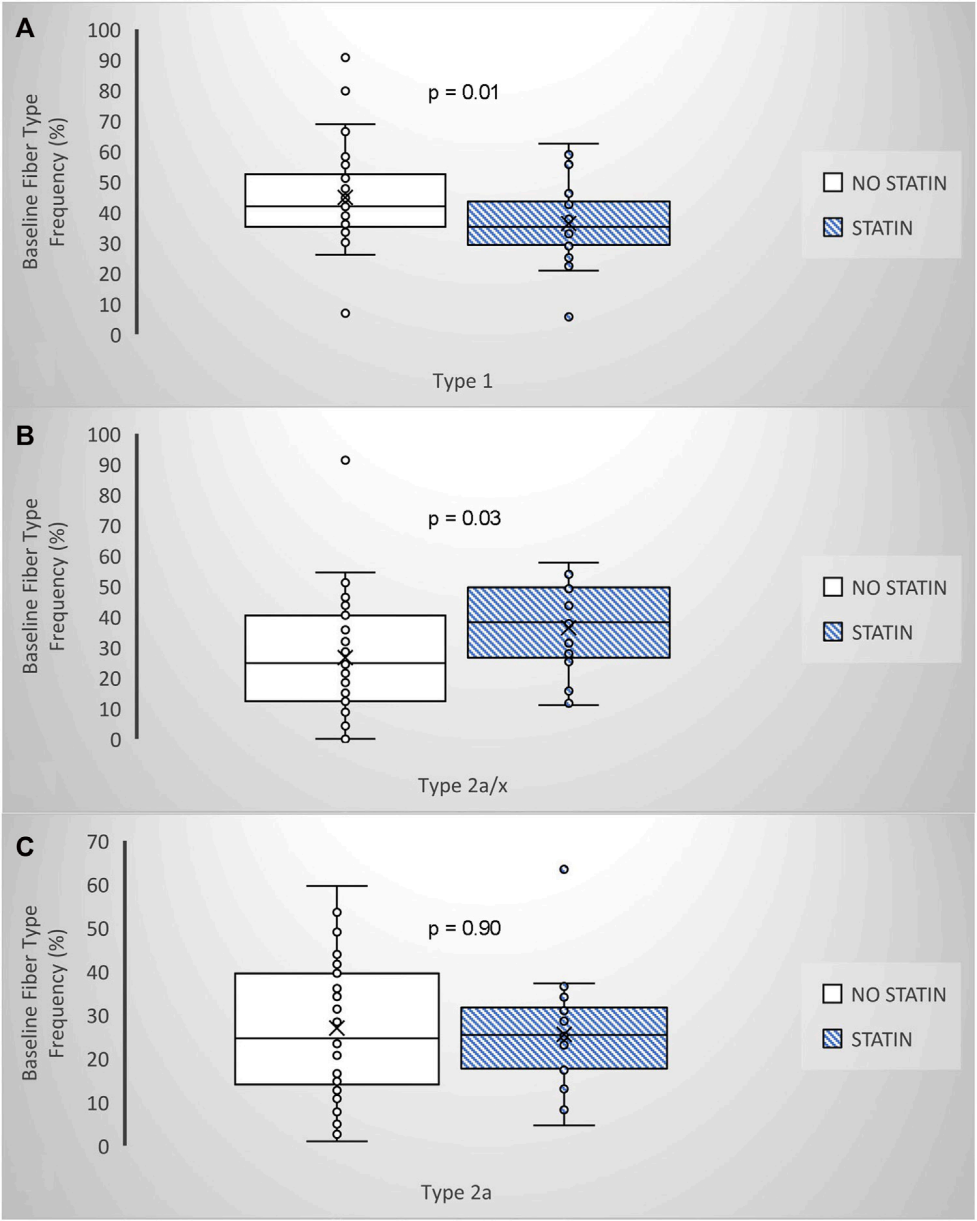
Statin therapy and fiber type frenquency prior to PRT amoung adults BOX and whisker plots show that those taking statins (*n* = 25) have **(A)** lower type 1,**(B)** higher 2a/x hybrid and **(C)** no difference in type 2a fiber frequency when compared to those not on a ststin (n-43). ANCOVA, adjusted for sexand physical activity. *p*-values are shown.

**FIGURE 2:**
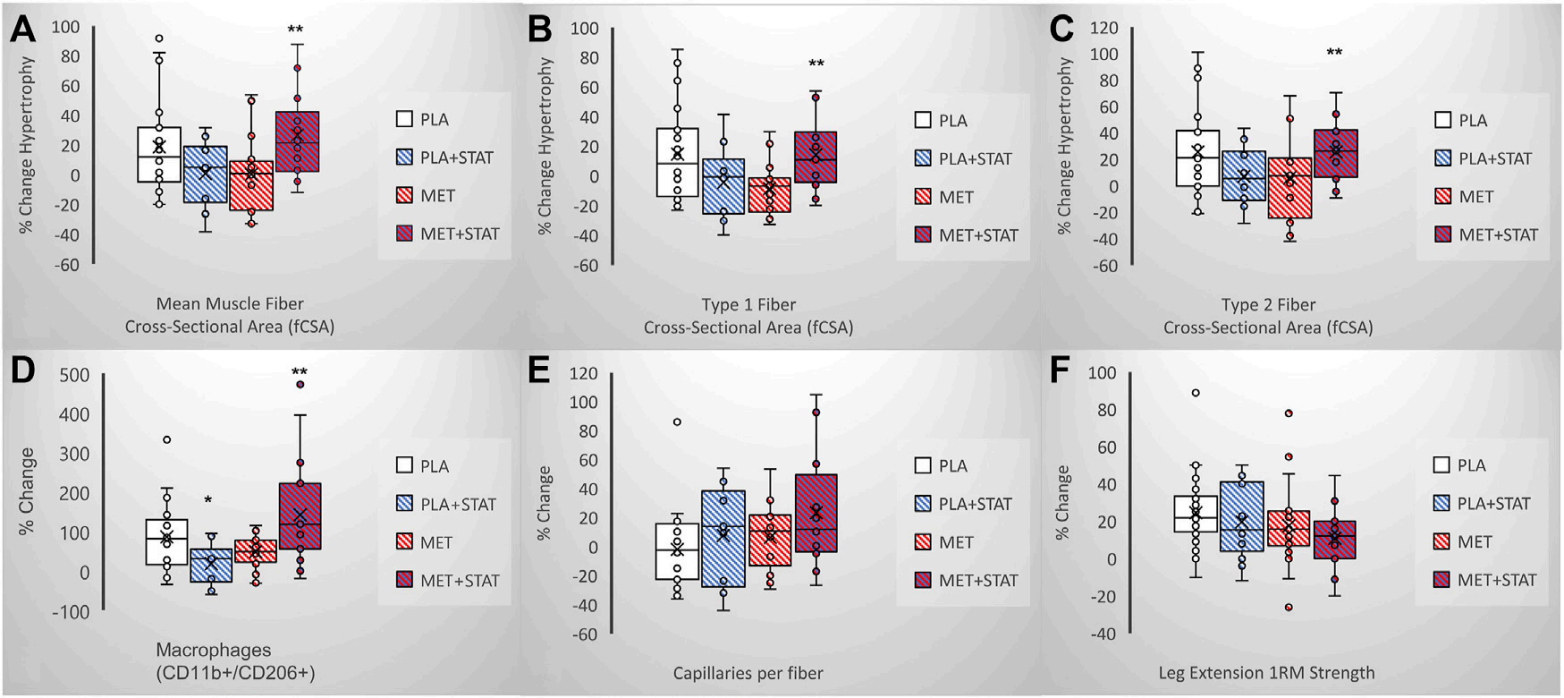
Statin effects on PRT outcomes with and without combined metformin. Box and whisker plots show **(A–C)** mean and fiber type-sspecific growth and **(D,E)** macrophage and capillary change differences between monotherapy versus combined metformin/stain use only (PLA + STAT, *n* = 10) does not affect mean fiber growth compared to placebo (PLA, *n* = 22), but combined metformin/stain therapy (MET + STAT, n = 14) significantly response when compared to metformin monotheraphy (MET, n-16) possibly due to siginificantly increased macrophages or increased capillary density (*p* = 0.09). Box and whisker plots **(F)** show that statin use only (PLA + STAT, *n* = 13) does not affect functional gains inleg extension strenght when compared to placebo (PLA, *n* = 31), but combined metformin/staintherapy (MET + STAT, *n* = 16) does not rescuse reduced strenght gains when compared to metformin monotherapy (MET, n-28) ANCOVA (adjusted for sex and stain use for muscle size and function, and sex, stain use, and anti-inflammatory medications for immune cells) *p*-values are shown.* denotes significance from placebo group,** denotes siginficance from metformin group.

**FIGURE 3:**
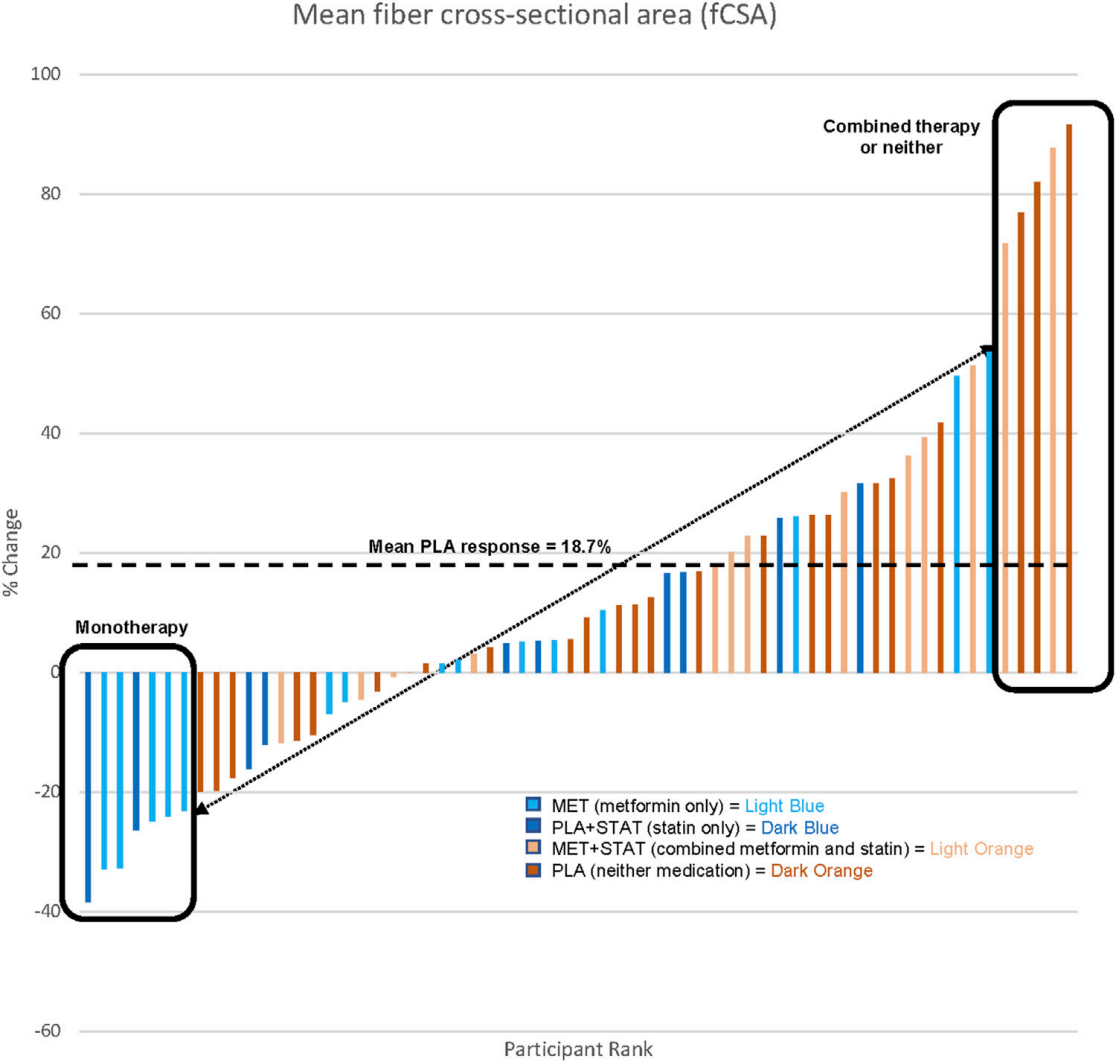
Anabolic heterogeneity amoung prescribed stain users (STAT,n-24) versus non-user (n-38) completing the MASTERD study who were randomized to recevied metformin (MET) or placebo (PLA), the average percent change in mean fiber cross section area (FCSA) for PLA participats is shown by thw dotted line and boxe represent extreme particle responces, FCSA was not determined for samples that were freeze damage or consisted of less than 300 fibers resulting in a total of *n* = 62.

Similar to the general population, approximately one-third of older adults in the MASTERS trial were taking a prescribed statin (*n* = 31/94; 11F, 20M). Thus, this current secondary analysis involved four groups; placebo only (*n* = 34, PLA; 23F, 11M), statin only (*n* = 14, PLA + STAT; 6F, 8M), metformin only (*n* = 29, MET; 17F, 12M), and metformin + statin (*n* = 17, MET + STAT; 5F, 12M). Since statin drugs all have different dosing ranges, we categorized each subjects’ statin dose as low-, moderate-, or high intensity, based on cholesterol lowering (Grundy et al., 2019). Prior to training, those on statin therapy had significantly fewer oxidative type 1 muscle fibers, more glycolytic type 2a/x hybrid fibers, and no difference in the frequency of type 2a fibers (Figures 1A–C), which were not associated with other factors such as reported physical activity (Table 1). This suggests an effect of statin therapy on resting muscle metabolism. Following PRT, overall muscle fiber growth appeared lower in statin users compared to the placebo only group affecting both type 1 and type 2 fibers, but this did not reach significance (PLA vs. PLA + STAT, *p*= 0.15, Figures 2A–C). In contrast, statin users randomized to metformin demonstrated a significantly higher increase in fiber cross-sectional area (fCSA) compared to metformin alone (MET vs. MET + STAT, *p*= 0.002), with significant growth occurring in both fiber types. Interestingly, we did not find significant effects of combined therapy on leg extension strength (PLA vs. PLA + STAT, *p*= 0.45) (MET vs. MET + STAT, *p*= 0.19) (Figure 2F). Stated differently, combined therapy seemed to ameliorate some of the metformin- or statin-mediated deficits in fCSA growth response to PRT, with more individuals responding similarly to those taking neither medication. However, muscle fiber size did not relate to strength gains, suggesting the medications may affect muscle quality. We did not find differences in baseline characteristics other than sex or medication use between our groups, and training adherence was also not different between groups (*p*= 0.41) when looking at individual heterogeneity in fCSA response to training (Figure 3).

**TABLE 1:** Baseline participant characteristics between medication groups.

Participant characteristics Mean ± SD (range) or N (frequency)	Metformin only (MET)	Statin only (STAT)	Combined therapy (MET + STAT)	Neither medication (PLA)	Whole model main effect between groups
Demographics					
Total N	29	14	17	34	94
Age (yrs)	70.0 ± 5.4 (64.8–91.2)	70.5 ± 4.3 (64.6–77.6)	69.9 ± 3.9 (65.3–80.3)	71.0 ± 4.6 (64.4–82.8)	0.84
Sex (% F)	17/29 F (59%)	6/14 F (43%)*	5/17 F (29%)*	23/34 F (68%)	0.05
BMI (kg/m^2^)	26.3 ± 2.9 (20.2–31.5)	27.4 ± 2.6* (22.5–30.3)	28.0 ± 3.4* (22.0–33.9)	24.9 ± 2.9 (18.56–30.3)	0.003
Medication use					
Statin Dose (%)					
High	N/A	2 (15%)	5 (29%)	N/A	0.49
Moderate		10 (70%)	11 (65%)		
Low		2 (15%)	1 (6%)		
# of hypertension drugs per person	0.4 ± 0.7 (0-2)	1.2 ± 1.0*^ (0-3)	1.1 ± 1.0*^ (0-3)	0.3 ± 0.6 (0-2)	0.0001
# of anti-inflammatories per person (NSAIDs/Fish Oil)	0.8 ± 0.9 (0-3)	0.9 ± 0.8 (0-2)	1.3 ± 1.0* (0-3)	0.4 ± 0.8 (0-3)	0.006
Exercise Adherence					
Sessions attended (%)	95.7	97.5	96.9	97.7	0.41
Baseline muscle mass, function, and physical activity					
Total N	28	13	16	31	88
DXA bilateral thigh muscle mass adjusted to femur length^a^	252.9 ± 43.9* (170.6–324.0)	253.5 ± 53.8 (187.1–329.2)	279.1 ± 41.2* (202.5–344.8)	223.1 ± 36.0 (160.6–290.9)	0.002
Leg extension 1 RM strength adjusted to thigh muscle mass^b^	0.01 ± 0.003 (0.004–0.02)	0.01 ± 0.002 (0.004–0.01)	0.01 ± 0.003 (0.005–0.02)	0.01 ± 0.003 (0.004–0.01)	0.11
PASE^c^ physical activity score	182.5 ± 75.5 (55-348.2)	169.0 ± 105.5 (56.4–492.5)	185.9 ± 50.2 (95.6–244.7)	147.5 ± 63.9 (52.5–386.5)	0.19
Percent change in function after resistance training					
Change in leg extension 1RM strength (%)	17.9 ± 19.6 (-27.8–79.8)	14.0 ± 18.5 (-5.8-42.1)	9.4 ± 14.8 (-19.2–37.8)	20.9 ± 19.3 (-10.5–91.4)	0.22

Baseline characteristics of participant group designations are shown. Differences were not found between those taking statins (STAT, vs. MET + STAT) and sex differences between groups accounted for BMI, and muscle mass differences seen.

a DXA, muscle mass in grams normalized to femur length in cm.

b One repetition-max (1RM) strength in kg normalized to thigh muscle mass in grams.

c Physical Activity Survey in the Elderly.

*Significantly different from PLA, group following post-hoc testing, ^ Significantly different from MET, group following post-hoc testing.

We previously reported that the increase in alternatively activated, M2-like (CD11b+/CD206+) macrophages was positively associated with changes in muscle stem cells, capillaries, and fCSA in response to PRT in MASTERS participants (Peck et al., 2022). In the present study, we found that statin use in placebo participants (PLA + STAT) reduced the increase in CD11b+/CD206+ macrophages (*p* = 0.03) following PRT (Figure 2D). By contrast, MET + STAT had a significantly higher increase in M2-like macrophages (*p*= 0.002) and trended toward greater increases in capillary density (Figure 2E, *p*= 0.09) compared to MET, potentially contributing to the augmentation in muscle fiber growth with combined drug treatment.

## Discussion

### Mechanisms of Action of Metformin and Statins

Both statins and metformin have complex mechanisms of action. Signaling pathways and downstream targets related to health benefits and side effects have been identified. Metformin, in terms of an anti-aging drug, is classified as a caloric restriction (CR) mimetic, because it can induce metabolic, physiological, and hormonal changes similar to reducing calorie intake. Metformin’s central role as an anti-diabetic agent is through its regulation of ATP production through modulation of the adenosine monophosphate-activated protein kinase (AMPK) pathway, resulting in reduced hepatic glucose output and overall glycemic improvement (Greenhill, 2018; Kaneto et al., 2021). AMPK also inhibits mammalian target of rapamycin complex 1 (mTORC1) signaling which may contribute to its anti-aging effects (Yu et al., 2021), but lowering mTORC1 activity in muscle by metformin may also limit protein synthesis in response to PRT (Goodman, 2019). Numerous potentially beneficial effects of metformin have been observed in anti-cancer, immunosuppression, cardiovascular, and neurodegenerative trials, suggesting a broad effect across multiple AMPK- independent mechanisms (Jin et al., 2016; Minamii et al., 2018; Saini and Yang, 2018; Hsu et al., 2021). Enhanced mitochondrial respiration and oxidative metabolism, reduced inflammatory response, reversal of endothelium dysfunction, autophagy induction, and reduced endoplasmic reticulum stress have all been noted and recent evidence suggests that metformin may also work in the intestine with effects on gut microbiota (Vaiserman et al., 2021).

Statins, on the other hand, work through the mevalonate pathway to inhibit a key enzyme (HMG-CoA) for sterol isoprenoid (cholesterol) production in the liver (Arefieva et al., 2018). Much like metformin, statins have become potential therapeutic targets for cancer, autoimmune, and neurological disorders, since sterol and non-sterol isoprenoids are involved in numerous metabolic and inflammatory processes (Buhaescu and Izzedine, 2007). Statin use is not well-tolerated in some individuals and can be associated with myotoxicity-induced muscle pain. Reduced mitochondrial content and function, disruptions in glucose and calcium metabolism, and inhibition of mTORC1 signaling resulting in protein breakdown via FOXO and atrogin-1 pathways are proposed mechanisms for statin intolerance (Bonifacio et al., 2015; Du Souich et al., 2017; Elsaid et al., 2017). A new report also shows metformin affects the FOXO-myostatin signaling axis causing muscle atrophy (Kang et al., 2021). With many overlapping and parallel mechanisms of action, the possible benefit of combination therapy has been proposed (van Stee et al., 2018).

### Metformin Effects on Resistance Training Outcomes in Older Individuals

Because approximately 30-35% of older adults do not gain muscle mass or strength in response to resistance exercise training (Long et al., 2017), we studied metformin for its anti-inflammatory properties on skeletal muscle macrophages in combination with PRT in the MASTERS Trial. We reported that overall muscle size and quality (density) gains determined by computed tomography (CT), as well as muscle mass by DXA, were significantly lower following PRT in the metformin compared to placebo group. However, this did not lead to significantly reduced strength gains consistent with findings in type 2 diabetics (Boulé et al., 2013). Metformin also blunted fiber type switching and RPS6 phosphorylation, a downstream target of mTORC1, associated with increased AMPK phosphorylation. Other work has shown that metformin use can impact insulin growth factor levels (IGF-1) following a single bout of resistance exercise (Moreno-Cabañas et al., 2022). In our subsequent follow-up analyses, we showed that metformin administration primarily affected those with unhealthy muscle lipid profiles prior to beginning their resistance exercise program (Long et al., 2021). Therefore, our work shows that as a monotherapy co-prescribed with exercise, metformin blunts desired PRT responses in healthy older adults. This is in line with the detrimental effects of metformin when combined with endurance exercise in healthy older individuals (Konopka et al., 2019). However, there are reported benefits of combined metformin and exercise in those with diabetes (Abdelbasset, 2021).

### Statin Use During Exercise

Statins have been evaluated for their effect on cardiovascular training adaptations and muscle performance. When combined with exercise, statins have been reported to negatively influence muscle strength and quality gains over time, increase the resting respiratory exchange ratio (suggesting increased glucose metabolism), and exacerbate the number of statin-related muscle complaints (Parker and Thompson, 2012). Their use also appears to impair gains in aerobic capacity and mitochondrial content (Miller and Thyfault, 2020). To the contrary, several studies have also found that statin use has no effect on aerobic exercise performance and may have benefit in certain performance-related tasks (Parker and Thompson, 2012). Limited evidence exists for statin effects in healthy older adults performing resistance exercise training. In these controlled trials, short-term statin therapy did not appear to impact the response to acute eccentric bouts of exercise (Panayiotou et al., 2013), or affect resistance training outcomes when combined with endurance exercise (Allard et al., 2021). On the other hand, Riechman et al. reported independent associations of statin use and lean mass increase following a 12-week high intensity resistance exercise training program in healthy older adults (Riechman et al., 2007). Thus, recommendations for continued use of statins during exercise have been made (Alturki et al., 2021). Nevertheless, the lack of randomized trials with healthy control groups, and differences in population-related medical history, statin dose, type of exercise, and performance measures chosen make evidence for statin use during exercise equivocal.

### Effects of Combined Metformin and Statin on Muscle Response to Progressive Resistance Training

To date, no study has examined the combined effects of metformin plus statins on exercise performance or muscle hypertrophy, especially in an at-risk group such as older individuals. Reanalysis of MASTERS data showed that combined drug therapy rescued the hypertrophic response, but had no effect on strength, following 14-weeks of PRT, associated with increased abundance of alternatively activated macrophages, which we have shown are correlated to increased muscle stem cell abundance, capillary density, and fSCA (Walton R. G. et al., 2019; Peck et al., 2022). It has been reported that statins may modify the susceptibility of skeletal muscle to membrane damage and injury in response to an acute exercise stress by exacerbating calcium and creatine kinase release (Parker and Thompson, 2012). This could possibly lead to a greater immune response seen in the current study. Furthermore, capillary accrual may facilitate growth both through increasing tissue oxygenation and immune cell infiltration into muscle. It is possible combination therapy may have a positive effect on this aspect of muscle adaptive response as we found a trend for increased capillary density following PRT with combined therapy (*p* = 0.09). Furthermore, metabolic dysfunction is identified as a potential mechanism underlying statin-induced muscle intolerance. We have previously reported that metformin induces skeletal muscle shifts in oxidative metabolism to resemble that of younger individuals (Kulkarni et al., 2018; Kulkarni et al., 2020b) which may help to balance a more glycolytic metabolic profile associated with statins. Similar results have been reported in mice where another metabolic modulator, trimetazidine, has been shown to restore an oxidative phenotype in muscle thereby alleviating statin-induced decrements in fCSA in response to exercise training (Song et al., 2018). Finally, recent work has implicated both metformin and statins as potential senolytics (Jadhav et al., 2013; Bennaceur et al., 2014; Kulkarni et al., 2020a; Hu et al., 2020), and it may be possible that these drugs work synergistically to limit senescent cell accumulation during exercise.

## Limitations and Remaining Challenges

This secondary analysis of our MASTERS study has limitations. The original study was not intended to examine the combined drug effects of metformin and statins, and hence the number of subjects in each group is limited. Those on statins were chronic statin users, but participants were taking different statins and different doses, although all were therapeutic doses. Of the 31 subjects taking statins, 24 were taking a lipophilic type (atorvastatin, simvastatin) while seven were taking a more hydrophilic type (pravastatin and rosuvastatin). We have analyzed these data as if all statins are the same irrespective of dose, but this may not be the case and the amount of time each participant was on a statin was not controlled. The addition of drug only, non-exercising control groups should also be incorporated into future study designs.

## Conclusion

In summary, several current investigations are aimed at anti-aging treatments to prolong healthspan with common medications such as metformin and statins taking precedent. Unfortunately, very little is known about these pharmaceuticals in the context of exercise adaptation. Collectively, our results suggest that taking either compound alone may cause deleterious consequences in exercising muscle, possibly due to shifts in muscle metabolism and inhibition of mTORC1 signaling. However, in our cohort, the hypertrophic adaptive response to PRT was rescued when metformin was combined with statin therapy. Thus, combination therapy may benefit the muscle microenvironment by preventing negative effects from monotherapy on cellular muscle features needed for more robust hypertrophy. Clinical trials focused on exercise-drug interactions need to be implemented in order to fully understand the impact on skeletal muscle adaptation to ensure that function and quality of life are maintained with age and benefits of exercise are maximized. In addition, exercise status should be considered by physicians when recommending these specific medications.

## Data Availability

The raw data supporting the conclusion of this article will be made available by the authors, without undue reservation.
